# Overexpression of JAG2 is related to poor outcomes in oral squamous cell carcinoma

**DOI:** 10.1002/cre2.267

**Published:** 2019-12-05

**Authors:** Kiichi Hatano, Chiemi Saigo, Yusuke Kito, Toshiyuki Shibata, Tamotsu Takeuchi

**Affiliations:** ^1^ Department of Oral and Maxillofacial Surgery Gifu University Graduate School of Medicine Gifu Japan; ^2^ Department of Pathology and Translational Research Gifu University Graduate School of Medicine Gifu Japan

**Keywords:** JAG2, Notch ligand, oral squamous cell carcinoma, prognosis

## Abstract

**Objectives:**

JAG2 is one of Notch ligands, which recently appear to exert various carcinogenesis. In the present study, we aimed to unveil the relation of JAG2 expression and clinicopathological features in oral squamous cell carcinoma (OSCC).

**Materials and Methods:**

We examined *JAG2* expression in OSCC plus adjacent nontumorous epithelia in eight patients. Ninety‐one OSCC tissue specimens were immunohistochemically stained with specific antibodies to JAG2. The immunoreactivities of JAG2 were correlated with clinicopathological factors, including the prognosis of patients. Chi‐square test, Kaplan–Meier survival, and Cox proportional hazard analysis were used to determine the statistical value of JAG2 expression in OSCC.

**Results:**

*JAG2* mRNA expression was much expressed in OSCC tissues compared with adjacent tissue specimens in five of eight patients. JAG2 immunoreactivity was found at invasion front in 31 of 91 OSCC. JAG2 immunoreactivity was significantly associated with age, less than 50 years old of patients (*P* = .048). Kaplan–Meier analysis demonstrated that the patients with JAG2 immunoreactvty have a short overall survival. With the Cox proportional hazard regression mode, the independent factors predictive of poor overall survival included JAG2 immunoreactivity (*P* < .05).

**Conclusions:**

The present findings suggest that JAG2 overexpression, especially at the cancer invasion front, has potential prognostic value.

## 1 INTRODUCTION

Oral squamous cell carcinoma (OSCC) is the common type of cancer in the world with increased incidence (Warnakulasuriya, 2009; Warnakulasuriya, 2010). Complete surgical resections at earlier stage is the best practice for patients with OSCC, whereas its overall 5‐year survival rate remained 50–60%(Siegel, Miller, & Jemal, 2017). Thus, there is a need to clarify the pathobiological mechanism of OSCC progression to develop new therapies.

Notch signaling pathway appeared to have diverse roles, oncogenic or tumor suppressor, in various carcinogenesis (Aster, Pear, & Blacklow, 2017; Yap et al., 2015). Activation of Notch pathway requires the binding of Notch receptors, designated Notch‐1 through Notch‐4, to surface membrane ligands, that is, DLL1, DLL3, DLL4, JAG1, and JAG2 in human (Kopan & Ilagan, 2009). Despite of recent progress, it is still not fully understood how these ligands interact with various Notch receptors in carcinogenesis. Furthermore, ligand‐dependent Notch activation required mono‐ubiquitination of cytoplasmic domain of ligands, followed by internalization of ligands to cytoplasm (Itoh et al, 2003; Takeuchi et al, 2005). Indeed, cytoplasmic localization of Notch ligands is frequently found in various cancer cells (He et al., 2016; Townsend et al., 2019; Zhang et al., 2014a).

One of Notch ligand, JAG2 appeared to exert several carcinogenesis, endometrial cancer (Townsend et al., 2019), colorectal cancer.(He et al., 2016), myeloma.(Ghoshal et al., 2009; Houde et al., 2004), pancreatic cancer (Mullendore et al., 2009), and breast cancer (Xing et al., 2011). In OSCC, few studies also suggested that JAG2 was overexpressed (Osathanon, Nowwarote, & Pavasant, 2016a; Zhang et al., 2011). Notably, blockade of the ligand–receptor interaction by a JAG2‐Fc fusion protein could inhibit the migration of pancreatic cancer cells (Hu et al., 2015). Moreover, arsenic trioxide inhibits JAG2 expression, thus impairing myeloma cell growth (Hu, Huang, Hong, Lu, & Zhu, 2013). This indicates that JAG2 could be a practical therapeutic target for various cancers.

Only a few studies have examined *JAG2* expression in OSCC (Osathanon et al., 2016a; Zhang et al., 2011). To the best of our knowledge, the prognostic value or the relationship between JAG2 expression and the clinicopathological factors remains unknown. To know the potential value of JAG2 as a molecular target for patients with OSCC, it is important to reveal the clinicopathological features of JAG2 expression in OSCC.

The aim of this study was to find the association between JAG2 expression and prognosis and clinicopathological factors of OSCC.

Regarding OSCC, there are still a few studies that examined the JAG2 expression (Osathanon et al., 2016a; Zhang et al., 2011). A few studies have suggested that JAG2 is also overexpressed in OSCC (Osathanon et al., 2016a; Zhang et al., 2011). However, to the best of our knowledge, the prognostic value or relation of JAG2 expression to clinicopathological factors remains unknown. To know the potential value of JAG2 as a molecular target for patients with OSCC, it is important to unravel the clinicopathological features of JAG2 expression in OSCC.

## 2 MATERIALS AND METHODS

### 2.1 Patient tissue specimens

After informed consent was obtained, surgically resected OSCC specimens and surrounding noncancerous tissues were stored frozen at −80°C for RNA extraction. In a retrospective study, we collected data on all surgically treated patients who were primarily diagnosed with OSCC. Archived pathological tissue specimens from 91 cases of OSCC were used in this study. Information of the patients with OSCC is shown. The present study was conducted in accordance with the ethical standards of the Declaration of Helsinki in 1975. The use of tissue samples and review of the clinical records were performed according to protocols approved by the Institutional Review Board of the Gifu University Graduate School of Medicine (specific approval number: 2018‐005, 2019‐157).

### 2.2 Quantitative real‐time reverse transcription PCR

Extraction of total RNA and synthesis of cDNA were performed with RNeasy Mini Kit (Qiagen, Valencia, CA) and Reverse Transcription Polymerase Chain Reaction (RT‐PCR) Kit (TaKaRa, Shiga, Japan) as previously described (Takao et al., 2017). Real‐time PCR was performed with a SYBR Green Reaction Kit according to the manufacturer's instructions (Roche Diagnostics, GmbH, Mannheim, Germany) on a LightCycler (Roche Diagnostics).

The following primers were used for PCR: *JAG2*, forward 5′‐TACCAACGACTGCAACCCTC‐3′; reverse 5′‐GCACTCGTCGATGTTGATGC‐3′; *GAPDH*, forward 5′‐GAAGGTGAAGGTCGGAGTC‐3′; reverse 5′‐GAAGATGGTGATGGGATTTC‐3′. The expression of each target gene was analyzed by the 2^–ΔΔCt^ method.(Livak & Schmittgen, 2001) using the LightCycler system. ΔCT values of genes were normalized to that of *GAPDH* for each triplicate set. The expression ratio of tumor tissue to nontumorous tissue, T/N, was calculated.

### 2.3 Immunohistochemical staining

All tissue specimens were obtained surgically, fixed in 10% buffered formalin, and embedded in paraffin. A tissue section, which represent the deepest invasion site of each case was immunohistochemically stained. The rabbit antibody to JAG2 (cat no. NBP1‐86337, 1:100) was purchased from Novus Biologicals (Littleton, CO). The tissues were immunostained with antibodies using the ImmPRESS™ polymerized reporter enzyme staining system (Vector Laboratories, Inc., Burlingame, CA, USA) as previously reported (Takeuchi et al., 2000).

### 2.4 Evaluation of immunohistochemical staining and statistical analysis

We evaluated the immunohistochemical staining results as a percentage of immunoreactivity in OSCC cells. The fraction of positive cells stained at the cancer invasion front was scored after examining three high‐power fields (400×) per tissue section for each case. The staining was considered as negative if less than 10% of invasive cancer cells exhibited immunoreactivity and as positive if over 10% did.

Curves for overall survival (OS) were drawn using the Kaplan–Meier method, and differences in the survival rates were compared using the log‐rank test. The relationship between the clinicopathological parameter was examined using the chi‐square test. Multivariate analyses of Cox proportional hazard model were also used to determine the significant prognostic factors of OS. A *P* value of <.05 was considered statistically significant.

## 3 RESULTS

### 3.1 *JAG2* mRNA expression in OSCC and neighboring tissues

**Figure 1:**
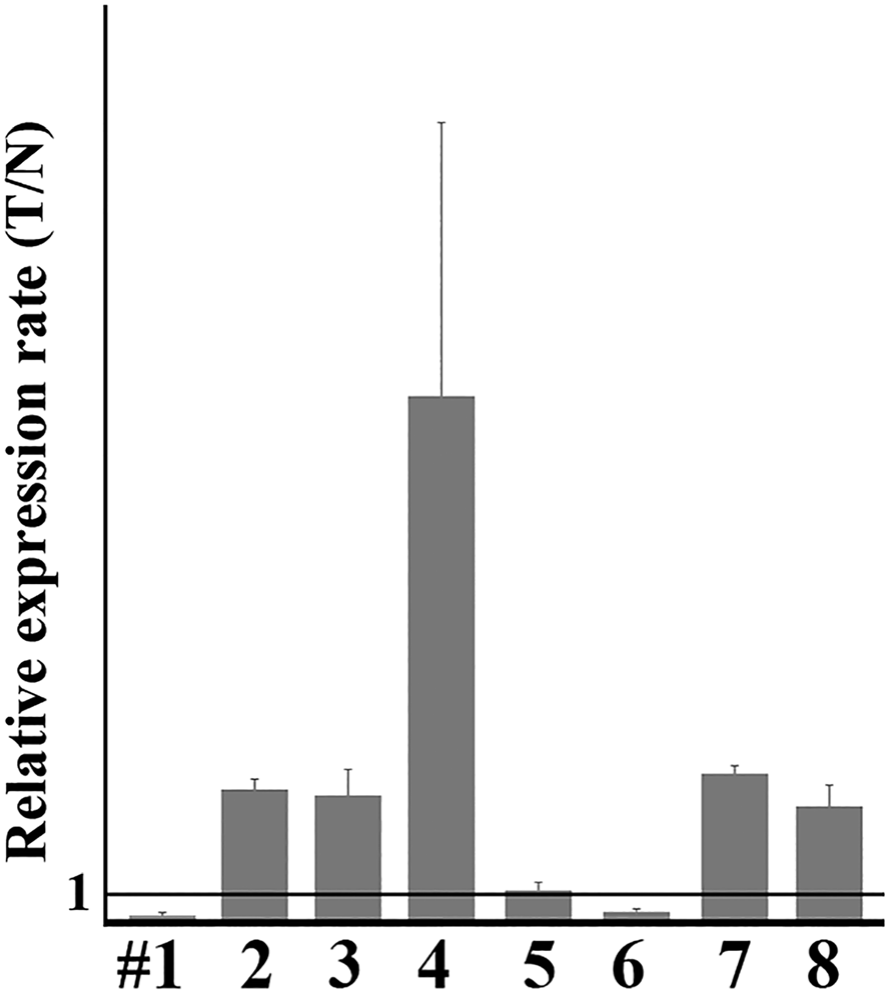
Relative *JAG2* mRNA expression ratio between tumor (T) and nontumorous (N) tissue specimens. *JAG2* mRNA level in OSCC (T) and paired nontumor tissues (N) measured by quantitative RT‐PCR. The ratio of concentration of *JAG2* mRNA of tumor/nontumor (T/N) is shown. Expression of *JAG2* mRNA in paired tumors and nontumorous tissue specimens was analyzed by triplicate and normalized to *GAPDH* expression. Note the specimens from patients #2, #3, #4, #7, and #8 in which *JAG2* mRNA was much expressed in tumor than nontumorous tissue (T/N ratio was over 1; mean and standard deviation are shown). Patients #4 and #8 were early forties and thirties, respectively

Results are summarized in Figure 1. Notably, *JAG2* mRNA was much expressed in OSCC tissues compared with the neighboring noncancerous tissues of five of eight patients. Notably, OSCC from two of two patients of relatively young age (patient #8 was early thirties, and patient #4 was early forties) exhibited robust *JAG2* mRNA compared with noncancerous tissues.

### 3.2 JAG2 expressions in OSCC tissue specimens in relation to clinicopathological parameter

**Figure 2:**
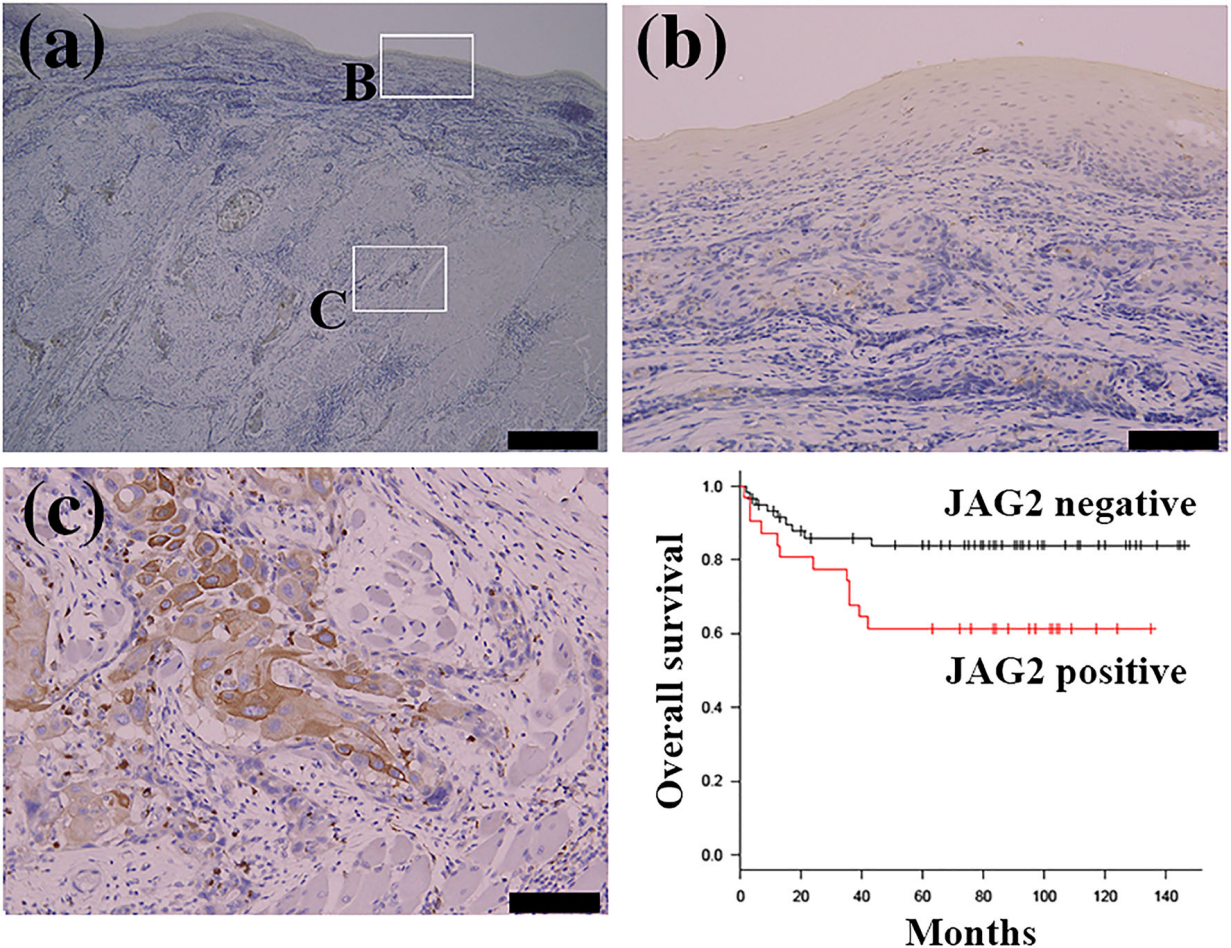
Representative immunohistochemical staining and overall survival curves according to JAG2 immunoreactivity at the invasion front of OSCC. Representative immunohistochemical staining using specific antibody to JAG2 (a). Weak or little JAG2 immunoreactivity was found in nontumorous oral epithelial cells or OSCC at superficial invasion site (b). In contrast, JAG2 immunoreactivity was found in cancer invasion front of OSCC (c). Bar indicates 400 μm (a) and 100 μm (b and c). The Kaplan–Meier method and differences in the survival rates were compared using the log‐rank test for univariate survival analysis (d). The overall survival rate of patients in the JAG2‐positive group was significantly lower than that of patients in the JAG2‐negative group (*P* = .0221 [<.05])

Clinicopathological characteristics of patients were summarized in Table 1. Representative results from the immunohistochemical staining are shown in Figure 2. Little immunoreactivities were found in nontumorous oral mucosa epithelial cells (Figure 2b). By contrast, JAG2 immunoreactivity was found in 31 of the 91 invasive cancer cell samples at the invasion front (Figure 2c).

**Table 1:** Summary of the clinicopathological characteristics of patients with OSCC

Characteristic	No. of patients (%)
Gender	
Male	46 (51)
Female	45 (49)
Age	
Average (years)	66.6
<50	14 (15)
≧50	77 (85)
Tumor location	
TON	35 (38)
LG	25 (27)
BM	16 (18)
UG	9 (10)
FOM	4 (4)
HP	2 (2)
Histology of SCC	
Well‐differentiated	63 (69)
Moderately differentiated	22 (24)
Poorly differentiated	6 (7)
Tumor T status	
T1	24 (26)
T2	43 (47)
T3	13 (14)
T4a	11 (12)
Tumor N status	
N0	59 (65)
N1	14 (15)
N2	10 (11)
N3	8 (9)
Tumor M status	
M = 0	91 (100)
M ≧ 1	0 (0)
Stage (UICC 8th Ed)	
I	21 (23)
II	27 (30)
III	15 (16)
IV	28 (31)
Radiotherapy/Chemotherapy	
None	60 (66)
Radio only	1 (1)
Chemo only	12 (13)
Radio + Chemo	18 (20)
Survival status	
Alive	62 (68)
Deceased	29 (32)
Disease‐associated death	21

*Note.* Information of the patents with OSCC is shown.

Abbreviation: OSCC, oral squamous cell carcinoma.

JAG2 expression was significantly related to age of patents, younger than 50 years old. By contrast, no significant correlation was observed between JAG2 immunoreactivity and other clinicopathological factors, that is, gender, histology, tumor size, status of nodal metastasis, and stages of patients (Table 2).

**Table 2:** Correction between JAG2 immunoreactivity and clinicopathological factors

Characteristic	JAG2 immunoreactivity		
	Negative = 60 (66%)	Positive = 31 (34%)	*P* value
Gender			
Male	27 (30%)	19 (21%)	
Female	33 (36%)	12 (13%)	.141
Age			
<50	6 (7%)	8 (9%)	
≧50	54 (59%)	23 (25%)	.048
Histology of SCC			
Well	39 (43%)	24 (26%)	
Moderately + poorly	21 (23%)	7 (8%)	.224
Tumor T stuts			
T1 + T2	41 (45%)	26 (29%)	
T3 + T4	19 (21%)	5 (5%)	.111
Lymph node metastasis			
No	39 (43%)	20 (22%)	
Yes	21 (23%)	11 (12%)	.876
Stage (UICC 8th Ed)			
I + II	29 (32%)	19 (21%)	
III + IV	31 (34%)	12 (13%)	.241
Perineural invasion			
No	55 (60%)	27 (30%)	
Yes	5 (5%)	4 (4%)	.489
Lymphovascular invasion			
No	28 (31%)	15 (16%)	
Yes	32 (35%)	16 (18%)	.876
Vascular invasion			
No	47 (52%)	22 (24%)	
Yes	13 (14%)	9 (10%)	.437
Disease‐specific death			
No	51 (56%)	19 (21%)	
Yes	9 (10%)	12 (13%)	.011

*Note.* Immunoreactivity of JAG2 associated with clinicopathological characteristics is shown. Chi‐square test revealed that JAG2 immunoreactivity was significantly related to age, less than 50 years old, and poor overall survival of patents with OSCC.

Abbreviation: OSCC, oral squamous cell carcinoma.

Kaplan–Meier method followed by the log‐rank test revealed that JAG2 immunoreactivity was correlated to poor OS of patients with OSCC (*P* = .0221; Figure 2d). Using multivariate analysis, JAG2 immunoreactivity appeared to be an independent marker of poor OS (*P* = .009, Cox proportional hazard regression model; Table 3).

**Table 3:** Cox multivariate analysis in OSCC

	Univariate	
	Hazards ratio (95% CI)	*P* value
Sex	1.863 [0.815, 4.262]	.14
Age (<50 vs. ≥50 years)	1.50 [0.450, 5.062]	.505
Histological differentiation (well‐differentiated vs. moderately and poorly differentiated)	1.247 [0.533, 2.914]	.611
T stage (T1‐2 vs. T3‐4)	1.500 [0.641, 3.508]	.35
Lymph node metastasis (no vs. yes)	4.175 [1.784, 9.77]	.0009
Lymphovascular invasion	3.957 [1.476, 10.61]	.006
Vascular invasion	2.838 [1.271, 6.338]	.01
UICC stage (I + II vs. III + IV)	3.075 [1.274, 7.425]	.0125
JAG2	2.633 [1.109, 6.25]	.0282
	Multivariate	
	Hazards ratio [95% CI]	*P* value
Lymph node metastasis (no vs. yes)	1.373 [0.277, 6.794]	.698
Lymphovascular invasion	2.218 [0.655, 7.509]	.2
Vascular invasion	1.065 [0.399, 2.846]	.9
UICC stage (I + II vs. III + IV)	2.296 [0.431, 12.230]	.33
JAG2	2.916 [1.177, 7.222]	.02

*Note.* Cox proportional hazard model demonstrated that JAG2 immunoreactivity was significantly correlated to poor overall suvival as well as lymphovascular invasion, nodal metastasis, and UICC stage (I + II vs. III + IV).

Abbreviation: OSCC, oral squamous cell carcinoma.

## 4 DISCUSSION

An earlier study demonstrated that JAG2 expression was highly correlated in tongue carcinoma tissues (Zhang et al., 2011). Although it is unclear, whether or to what extent the study examined OSCC, Wenyue et al. reported that *JAG2* mRNA was significantly overexpressed in head and neck cancer compared with those in normal mucosa using Affymetrix HuEx 1.0 GeneChip analyses (Sun et al., 2014). In silico study based on Gene Expression Omnibus database highlighted *JAG2* as up‐regulated gene in OSCC (Osathanon, Nowwarote, & Pavasant, 2016b). The present study confirmed the overexpression of JAG2 expression at mRNA and protein levels in a part of OSCC specimens. As demonstrated in Figures 1 and 2, OSCC cells much expressed JAG2 compared with noncancerous epithelial cells at least in a part of patients. Taken together with previous reports, it is apparent that overexpression of JAG2 is a gain phenotype of a part of OSCC.

In addition to OSCC, JAG2 is overexpressed in many malignant tumors, including myeloma, (Ghoshal et al., 2009; Houde et al., 2004) pancreatic cancer, (Mullendore et al., 2009) and breast cancer(Xing et al., 2011). However, the prognostic value of JAG2 expression depends on the cancer type. Kang et al. reported that JAG2 overexpression was associated with a favorable outcome in patients with gastric cancer (Kang et al., 2012). Kirana et al. also reported that a strong expression of JAG2 in tumor cells was associated with a good colorectal cancer‐specific survival (Kirana et al., 2019). Pancewicz‐Wojtkiewicz et al. reported no statistically significant associations between survival and JAG2 expression in nonsmall cell lung carcinoma (Pancewicz‐Wojtkiewicz et al., 2017). In contrast, JAG2 expression is associated with a poor prognosis in patients with hepatocellular carcinoma (Zhang et al., 2014b) and urinary bladder cancer(Li et al., 2013).

The present result indicated that JAG2 was expressed in cancer invasion front of 31 of 91 OSCC tissue specimens. To our knowledge, this is the first study that unveiled that JAG2 expression was related to poor OS of patients with OSCC. Notably, JAG2 expression was significantly related to age, younger than 50 years old, in our OSCC tissue specimen series. OSCC primarily occurs in older age group. However, in the recent years, incidence of oral cancer in young people has been on rise worldwide (Abdulla et al., 2018). The pathogenesis of OSCC affecting young patients remains largely unknown, and the well‐known etiological factors for oral cancer, tobacco, and alcohol use are believed to play a minor role in the carcinogenesis of the neoplasm, suggesting that the etiology and the molecular basis of OSCC may differ between younger and older patients (Abdulla et al., 2018, Dos Santos Costa et al, 2018). Overexpression of JAG2, largely in cytoplasm in a part of OSCC at cancer invasion front, might be linked to the novel molecular mechanism, which was responsible for oral carcinogenesis at relative young age. However, it needs further extensive studies to unveil the molecular and pathobiological mechanism of JAG2‐mediating oral carcinogenesis.

In conclusion, the present findings indicate that one of Notch ligands, JAG2, was significantly overexpressed at invasion front of OSCC in one third of specimens with relation to poor prognosis.

## CONFLICT OF INTEREST

None declared.
